# Heterogeneity and permeability estimation of pore-throat structure at different scales in deep tight sandstone reservoirs: A case study of Paleogene Hetaoyuan Formation in Anpeng area, Nanxiang Basin, China

**DOI:** 10.1371/journal.pone.0314799

**Published:** 2024-12-27

**Authors:** Yan Zhu, Yunfei Yang, Yuheng Zhang, Lin Liu, Hengquan Li, Qin Sang

**Affiliations:** 1 Exploration and Development Research Institute of Sinopec Henan Oilfield Branch, Nanyang, China; 2 School of Geoscience and Technology, Southwest Petroleum University, Chengdu, China; China University of Mining and Technology, CHINA

## Abstract

Clarifying the pore-throat size and pore size distribution of tight sandstone reservoirs, quantitatively characterizing the heterogeneity of pore-throat structures, is crucial for evaluating reservoir effectiveness and predicting productivity. Through a series of rock physics experiments including gas measurement of porosity and permeability, casting thin sections, scanning electron microscopy, and high-pressure mercury injection, the quality of reservoir properties and microscopic pore-throat structure characteristics were systematically studied. Combined with fractal geometry theory, the effects of different pore throat types, geometric shapes and scale sizes on the fractal characteristics and heterogeneity of sandstone pore throat structure are clarified. On this basis, the estimation model of tight sandstone permeability was established. The results indicate that the reservoir physical properties in the study area are poor, the pore types are mainly dissolved pores, and the pore size is mainly distributed in the nano to submicron range. The fractal dimension fitting curve obtained based on the non-wetting phase model has obvious turning points, indicating that the pore-throat structure has multi-scale characteristics. The turning point of fractal dimension divides the pore-throat structure of tight sandstone into large-scale pore-throats with good connectivity (reticular or beaded pore-throats) and small-scale pore-throats with poor connectivity (dendritic or capillary pore-throats), indicating that tight sandstone has binary pore structure characteristics. The geometry of large-scale pore-throat is complex, which is difficult to meet the self-similar characteristics, with the average fractal dimension is 3.72. The small-scale pore-throat morphology is close to the capillary and has obvious fractal characteristics, with the average fractal dimension is 2.22. There are many small pores and micropores in the reservoir, and the pore volume has a significant positive correlation with the total porosity of the rock, but the contribution to the permeability is low. The development degree of large-scale pore throat is an important factor affecting the physical properties of tight sandstone. The turning point radius of fractal curve and the comprehensive fractal dimension can be used as good indicators for permeability estimation.

## 1 Introduction

With the continuous increase in global energy demand and the rapid reduction of recoverable resources in conventional oil and gas reservoirs, unconventional resources such as tight sandstone oil and gas have been paid more and more attention by the oil and gas industry [1, 2]. China has a huge potential for tight sandstone gas resources, which are widely distributed in the Upper Paleozoic in the Ordos Basin, the Xujiahe Formation in the Sichuan Basin and the Kuqa Depression in the Tarim Basin. The total amount of tight sandstone gas geological resources of the three groups accounts for 75% of the country’s total, and they are the main strata for tight gas exploration and development in China [3, 4]. Tight sandstone reservoirs usually have the characteristics of poor physical properties, large difference in pore size distribution, strong heterogeneity of pore-throat structure, and low natural productivity, which restrict the exploration and development of such resources [5, 6]. The characteristics of sandstone pore size, pore-throat connectivity and pore-throat network heterogeneity directly determine the occurrence and migration of tight gas during reservoir formation [7, 8], as well as the flow resistance and water absorption capacity in production and development [9], which are important factors to control the production and ultimate recovery rate of tight gas wells. Furthermore, the complex pore structure can lead to high immobile water content (bound water), resulting in significant differences in the relative content and occurrence characteristics of movable fluids, which brings difficulties to the identification of fluid properties and the calculation of producing reserves [10, 11].

At present, the experimental methods for characterizing pore throat structures mainly include casting thin sections (CTS), scanning electron microscopy (SEM), CT scanning (X-CT), high-pressure (HPMI)/constant velocity (CVMI) mercury intrusion, *N*_2_ adsorption/desorption, and nuclear magnetic resonance (NMR) [12–15], which characterize pore throat structures through direct observation or indirect measurement. However, due to complex sedimentation and diagenesis, tight sandstone reservoirs have a wide range of pore size distribution (nanometer micron scale), complex pore throat network structures [16], and single experimental methods and conventional Euclidean geometry theories are difficult to accurately and quantitatively characterize pore structures [16]. The fractal geometry theory proposed by Mandelbrot is an effective method for characterizing the heterogeneity of porous media [17]. The fractal geometry theory is an effective method for characterizing the complex and irregular pore structure of porous media [18, 19]. It not only provides new ideas and methods for studying the complex micro pore throat structure of porous media in oil and gas reservoirs, but also builds a bridge between studying reservoir physical properties and micro pore throat structure [20, 21].

Scholars have proposed many fractal dimension calculation models based on different testing methods to quantitatively characterize the pore-throat structure characteristics of tight sandstone reservoirs, including thermodynamic models, Li models, three-dimensional capillary models, Sierpinski models, FHH models, and Menger fractal models [22–24]. Due to the different assumptions of each fractal model, the fractal dimensions calculated by these models vary greatly and represent different physical meanings [25, 26]. The fractal model based on HPMI has widely used for quantitative characterization of pore structure in sandstone reservoirs. The models based on HPMI to characterize the fractal characteristics of internal pores in porous media usually include wetting phase model and non-wetting phase model [27–29]. It is generally believed that the non-wetting phase model is more suitable for characterizing the pore-throat structure of low permeability tight sandstone. The fractal curve of this method will exhibit obvious segmented fractal characteristics, which also indicate the strong heterogeneity of pore-throat structure and the complexity of pore size distribution [30]. In addition, many studies have proposed that the fractal dimension of pore-throat structure (three-dimensional Euclidean space) range from 2 to 3. The increase in fractal dimension value also represents the irregularity of the pore-throat structure and the increase of surface roughness. It also reflects that the pore shape of tight sandstone changes from regular to complex, thus increasing the specific surface and hindering the migration of pore fluid in hydrophilic rocks [31, 32]. Although previous researchers have conducted numerous meaningful studies on the fractal characteristics of pore structure in low-permeability tight sandstone reservoirs, the feature parameters obtained based on HPMI can characterize the pore-throat structure characteristics of sandstone from different perspectives. However, the relationship between pore size distribution, pore-throat geometry, pore size, and reservoir properties cannot be quantitatively characterized, and the previous pore-throat scale division standards based on fixed pore size cannot meet the characteristics of tight sandstone reservoirs, such as large pore-throat scale span and complex pore-throat structure. Nevertheless, the scale division of different pore-throats not only plays an important role in evaluating reservoir properties, but also serves as an important means of characterizing pore-throat combinations of different types and geometries, which is crucial for predicting permeability and evaluating fluid mobility in tight sandstone reservoirs.

The deep tight sandstone reservoir of the IX group of the third member (Eh_3_^Ⅸ^) of the Hetaoyuan Formation in the Anpeng area of Biyang Sag in the southeast of the Nanxiang Basin experiences near-source rapid accumulation with superior reservoir-forming conditions [33, 34]. Multiple wells have been fractured and industrial gas flows have been observed, indicating good exploration and development potential. Compared with the tight sandstone in the shallow acidic coal-bearing strata, the deep tight sandstone in this area is deeply buried, with a higher degree of alkaline diagenetic evolution. Strong compaction, pressure dissolution, and cementation filling are more pronounced, resulting in poor physical quality, complex pore structure, unclear gas-water relationship, and high bound water saturation in sandstone reservoirs. Many scholars have mainly focused on sedimentary characteristics, fan delta sand body configuration, reservoir logging evaluation, reservoir characteristics, and main controlling factors, but there are few studies on the quantitative characterization of heterogeneity and permeability estimation of pore throat structure of tight sandstone reservoirs in this area. This study takes the Eh_3_^Ⅸ^ tight sandstone reservoir in Anpeng area as the research object. Based on the previous studies on the sedimentary geology and reservoir characteristics of the target layer in the area, the CTS and SEM are used to visually identify the type and structure of pore-throats. X-ray diffraction testing is used to analyze the geometric shape of pore throats and the occurrence characteristics of clay minerals in the pore network. The pore-throat characteristic parameters and complexity of pore-throat structure are obtained through the HPMI. Based on the fractal geometry theory, different types of pore-throat structures were classified by integrating reservoir pore-throat connectivity, pore size distribution, and fractal dimension. The pore-throat structure characteristics of tight sandstone reservoir and its relationship with reservoir properties and fractal dimension were systematically studied. On this basis, the relationship between the pore throat radius corresponding to the turning point of the fractal curve and the pore structure parameters was discussed. The relationship between the geometric shape characteristics and fractal dimension of the binary pore structure of tight sandstone was systematically studied, and it was clarified that there are significant differences in pore throat types and geometric shapes at different scales, which are key factors affecting the permeability of reservoirs and the strength of pore throat structure heterogeneity. A more accurate improved permeability prediction model was established by combining the turning point radius of fractal curves and the comprehensive fractal dimension, which has better performance than conventional models. The research results provide a theoretical basis for the sweet spot evaluation and productivity prediction of ultra-low permeability tight sandstone in terrestrial alkaline formations.

## 2 Geologic setting

**Fig 1:**
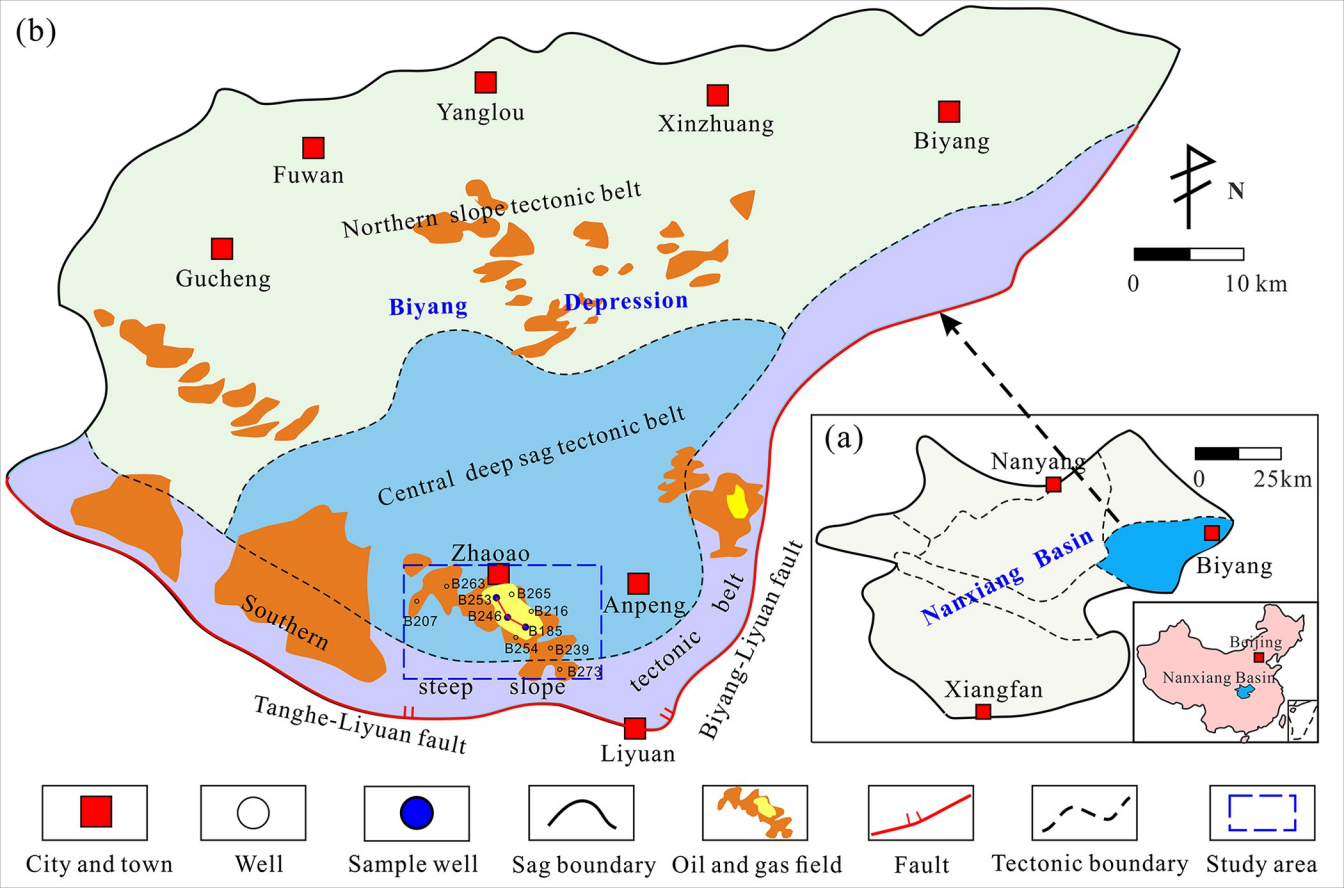
The location map of structure zone in the study area.

The Nanxiang Basin is located in eastern China and is the largest Cenozoic faulted continental petroliferous basin in the East Qinling-Dabie orogenic belt (Fig 1A). The Biyang Depression is located in the eastern part of the Nanxiang Basin and is a small intermountain inland faulted lake basin of the Mesozoic and Cenozoic eras. The Anpeng structure in the research area is located in the southeast steep slope zone of the Biyang Depression, which is a nose shaped structure with a northwest southeast dip. The main area of oil and gas is 3.3 km^2^ (Fig 1B). The Paleogene in the Anpeng area developed from bottom to top, including the Yuhuangding Formation, Dacangfang Formation, Hetaoyuan Formation, and Liaozhuang Formation. The Hetaoyuan Formation is a key exploration layer and can be further subdivided into the He-1, He-2, and He-3 Member [35]. The third section of the core is rich in oil and gas resources, and can be further divided into 9 third order sequences (I-IX) based on sedimentary cycles and sand body development characteristics. The deep strata in the Anpeng area mainly refer to the seventh, eighth, and ninth sand formations in the lower sub section of the third core with a burial depth greater than about 3000m, which are key exploration layers in the area. The Eh_3_^Ⅸ^ sand formation is an important tight gas production layer with a thickness of about 300 meters, mainly composed of dark gray mudstone, gravel, sandstone, and gray brown mudstone dolomite. Eh_3_^IX^ mainly develops fan delta front subfacies, with relatively stable lateral distribution of distributary channel microfacies and estuarine bar microfacies. The reservoir sand bodies are widely stacked and distributed, which is conducive to the development of high-quality sandstone reservoirs.

## 3 Samples and methods

### 3.1 Samples and experimental methods

This study collected 14 tight sandstone samples from three key production wells in the Anpeng area (Table 1). The lithology is dark gray and gray fine-medium grained sandstone, with the sampling depth is 3300–3500 m. According to research needs, the rock core will be uniformly processed into a uniform cylinder of 25 mm x 50 mm. Then, the corresponding physical property tests, cast thin section (CTS) analysis, scanning electron microscopy (SEM) observation, and high-pressure mercury intrusion (HPMI) experiments were carried out.

**Table 1:** Pore-throat structure parameters measured by HPMI.

Samples	Well	Depth (m)	*Φ* (%)	*K* (×10^−3^ μm^2^)	*RQI*	*FZI*	*P*_d_ (MPa)	*r*_a_ (μm)	*r*_50_ (μm)	*S*_max_ (%)
S1	B185	3452.80	3.70	0.0267	0.027	0.69	5.98	0.032	0.043	88.4
S2	B185	3460.10	2.89	0.0184	0.025	0.84	2.39	0.054	0.043	75.7
S3	B246	3460.15	3.61	0.0172	0.022	0.58	2.39	0.043	0.110	75.6
S4	B246	3460.20	3.85	0.0349	0.030	0.75	1.50	0.066	0.077	75.8
S5	B246	3460.20	3.91	0.0519	0.036	0.89	0.94	0.059	0.126	80.1
S6	B246	3460.40	1.46	0.0020	0.012	0.78	9.48	0.010	0.011	84.4
S7	B253	3339.70	1.62	0.0050	0.028	0.52	3.77	0.027	0.045	92.5
S8	B253	3338.80	3.54	0.0513	0.038	1.03	0.59	0.059	0.176	87.6
S9	B253	3338.35	2.93	0.0472	0.040	1.32	0.95	0.074	0.141	82.4
S10	B253	3338.90	4.04	0.0230	0.024	0.56	3.76	0.034	0.075	85.0
S11	B253	3460.50	3.54	0.1073	0.055	1.49	0.94	0.095	0.139	80.3
S12	B253	3460.60	4.21	0.1591	0.061	1.39	1.50	0.127	0.166	92.4
S13	B253	3460.70	3.30	0.0224	0.026	0.76	2.39	0.061	0.078	73.0
S14	B253	3460.75	2.78	0.0117	0.020	0.71	3.78	0.055	0.072	81.3

Abbreviations: *Φ*, porosity; *K*, permeability; *P*_d_, threshold pressure; *r*_a_, average pore-throat radius; *r*_50_, median pore-throat radius; S_max_, maximum mercury saturation

#### 3.1.1 Physical properties、CTS and SEM

The samples were washed and dried (100°C, 24h). According to the industry test standard SY/T 5336–1996, the porosity and permeability of all samples were tested by CMS-300 gas porosity and permeability measuring instrument. The core was cut and polished to make 30–35 μm rock slices, and inject blue epoxy resin into the pore space of the rock slices under vacuum, and then the pore type and mineral characteristics were observed by polarizing microscope (Zeiss Axio Imager A1M). The rock slices were coated with a layer of gold and palladium conductive film, and the pore-throat geometry was analyzed by XL-30 scanning electron microscope.

#### 3.1.2 HPMI

According to China standard GB/T 29172–2012, the samples were dried to constant weight, and then the mercury injection test was carried out by the AutoPore IV 9500 mercury porosimeter. The maximum pressure of the instrument was 221 MPa, and the corresponding pore-throat size was about 3.3 nm. According to the HPMI test results, a series of parameters that can effectively characterize the pore-throat structure characteristics of sandstone reservoir can be obtained, including displacement pressure (*P*_d_), maximum pore throat radius (*r*_d_), median pressure (*P*_50_), median radius (*r*_50_) and maximum mercury saturation (*S*_max_) (Table 1). The relationship between capillary pressure (*P*_c_) and pore-throat radius (*r*) is shown in Eq 1, which can clarify the relationship between the mercury saturation and the corresponding pore-throat radius under different pressures, thus characterizing the pore-throat structure characteristics [36].

$$P_{c}=\frac{2\sigma \cos\theta }{r}$$ (1)

where *θ* is the contact angle (°) and *σ* is the interfacial tension (N/m).

### 3.2 Methodology

#### 3.2.1 Flow unit index

The flow zone index (*FZI*) is a characteristic parameter that characterizes the heterogeneity of reservoir pore structure, and the *FZI* based reservoir flow unit division method is widely used [37]. Carman proposed the Kozeny Carman equation and defined the reservoir quality factor (*RQI*) by integrating parameters such as pore permeability, capillary pressure, rock tortuosity, and particle specific area to characterize the microscopic characteristics of rocks [38]:

$$RQI=0.01\pi \sqrt{\frac{K}{\phi_{e}}}$$ (2)

Normalized porosity(*Φ*_z_):

$$\phi_{z}=\frac{\phi_{e}}{1-\phi_{e}}$$ (3)

Combining Eqs (2) and (3):

$$FZI=\frac{1}{\sqrt{F_{s}}\tau S_{gv}}=\frac{RQI}{\phi_{Z}}$$ (4)

Take the logarithm of both sides of Eq (4):

$$\mathrm{lg}RQI=\mathrm{lg}\phi_{z}+\mathrm{lg}FZI$$ (5)

where *K* is the horizontal permeability (×10^−3^μm^2^), *Φ*_e_ is the helium porosity (%); *F*_s_ is the shape factor; *τ* is Tortuosity; *S*_gv_ is the specific surface area (μm^-1^).

#### 3.2.2 Fractal theory

Fractal refers to a type of complex pattern with similar structure between the local and the whole. Many studies have shown that the pore-throat space of rock has good fractal characteristics. The fractal dimension calculated based on the HPMI of sandstone samples can quantitatively characterize the complexity and heterogeneity of pore-throat structure. The commonly used calculation models include wetting phase model and non-wetting phase model [39]. The tight sandstone reservoirs in the study area generally develop small pores (<0.1 μm), and the reservoir seepage capacity is poor. Mercury is also difficult to enter micropores or ’dead’ pores under high pressure. However, the fractal dimension calculation model based on the wetting phase includes these micropores, resulting in errors in the calculation results. Therefore, this study uses the non-wetting phase saturation model to calculate the fractal dimension of the pore structure. According to the fractal theory, the pore-throat distribution of sandstone reservoirs has fractal characteristics, and the relationship between the number of pore throats *N*(r) and the pore throat radius (*r*) is as follows:

$$N(r)\propto r^{-D_{f}}$$ (6)

where ∝ representing ‘proportionality’ and *D*_f_ is the fractal dimension.

Assuming that the rock pore-throat is a capillary model, then *N*(r) can be expressed as:

$$N(r)=\frac{V_{Hg}}{\pi r^{2}l}$$ (7)

where *l* is the length of the capillary and *V*_Hg_ is the cumulative mercury volume when the pore-throat radius is *r*.

Combining Eqs (1), (6) and (7):

$$S_{Hg}=\alpha {P_{C}}^{-(2-D_{f})}$$ (8)

where *S*_Hg_ is the mercury saturation and α is a constant.

By taking the logarithm of both sides of Eq (8):

$$\log S_{Hg}=(D_{f}-2)\log P_{C}+\log\alpha$$ (9)

According to Eq (9), the fractal dimension that characterizes the complexity of rock pore structure can be calculated by plotting the double logarithmic coordinate plots of *S*_Hg_ and *P*_c_, and by fitting the slope of the equation. Essentially, the fractal dimension of three-dimensional porous media is generally within the range of 2–3 due to the influence of surface geometric irregularity and roughness.

Previous studies have shown that when the pore-throat structure is complex and there are significant differences in pore size, due to the simplification of the pore-throat structure (capillary pore-throat) by the existing model, the calculated fractal dimensions of large scale and small-scale pore-throats are quite different. Therefore, the porosity ratio of large pores and small pores and the permeability contribution of corresponding pore space are counted respectively. Then the total fractal dimension *D*_w_ of the whole pore space is obtained by weighted average of the porosity of different scale pore space.


$$D_{w}=\frac{D_{1}\times \Phi_{1}+D_{2}\times \Phi_{2}}{\Phi_{1}+\Phi_{2}}$$ (10)

## 4 Results

### 4.1 Reservoir petrophysical properties

#### 4.1.1 Petrological characteristics

**Fig 2:**
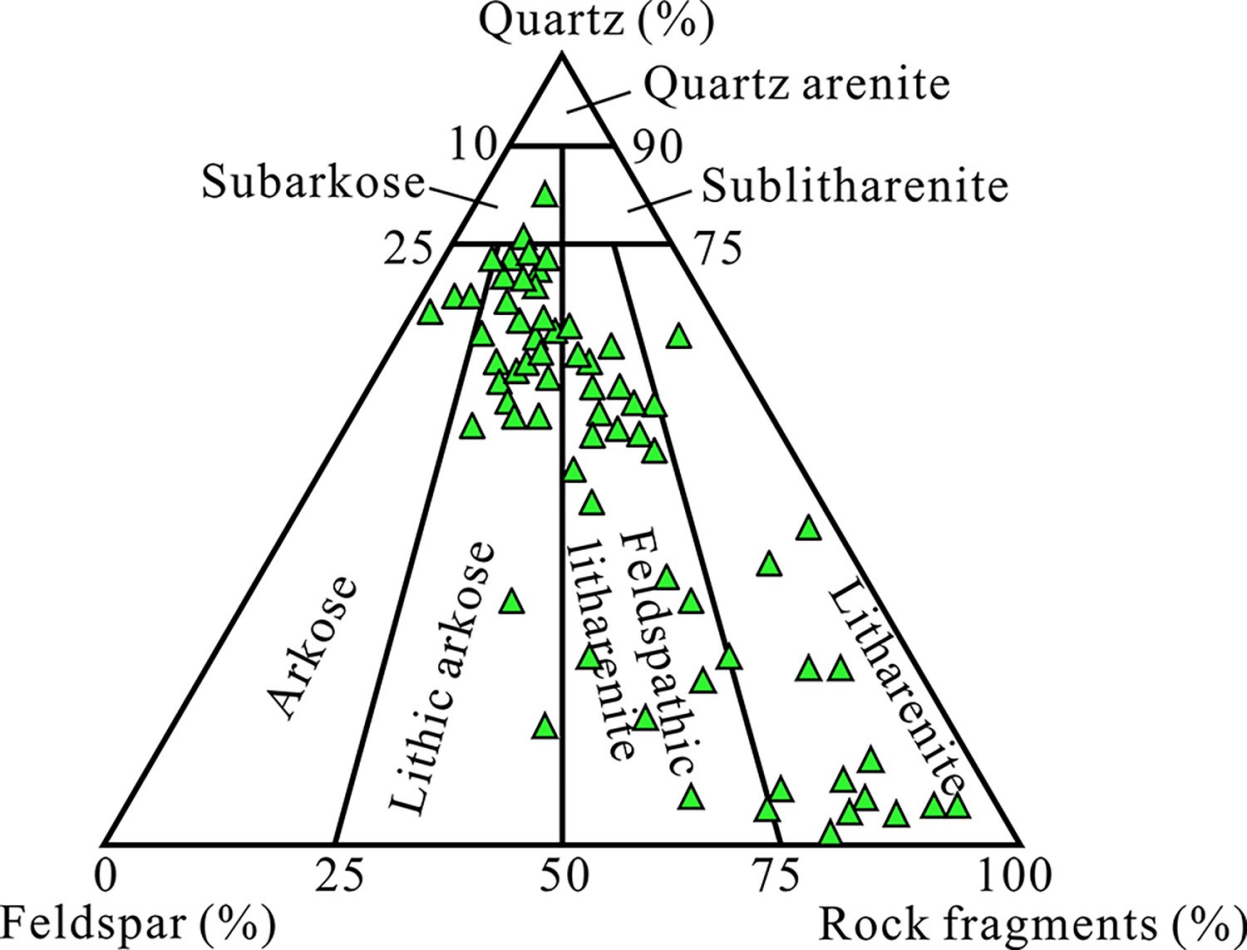
Triangular diagram of sandstone rock types of Eh3Ⅸ Formation in Anpeng area.

According to the analysis of core thin section data, the Eh_3_^Ⅸ^ deep tight sandstone reservoir is mainly composed of lithic feldspar sandstone, feldspar lithic sandstone, and lithic sandstone (Fig 2). The detrital components are mainly composed of quartz, feldspar, calcite, and clay minerals. Among them, quartz particles are mainly single crystal and microcrystalline quartz, with a high content and a large range of variation, ranging from 37.93% to 79.07% (averaging 47.3%). The second is rock debris, mainly composed of slate and marble, with a content distribution of 5.98%–46.64% (averaging 33.2%). Feldspar undergoes dissolution and is replaced by authigenic clay, with a relatively low content of 12.84%-27.79% (averaging19.5%). The roundness and sorting of reservoir rocks are poor, with the particle size mainly composed of fine sand and silt, containing some medium sand and gravel. The filling materials mainly consist of carbonate and clay matrix cementitious materials with low matrix content. The cementitious materials mainly consist of embedded crystals and filling structures, and the cementation type is mostly porous cementation. The clay mineral content is relatively low (averaging 1.81%), mainly consisting of illite (40%–61%, averaging 47.6%), chlorite (34%–55%, averaging 45.2%), and a small amount of chlorite/montmorillonite clay (2%–15%, averaging 7.2%).

#### 4.1.2 Porosity and permeability

**Fig 3:**
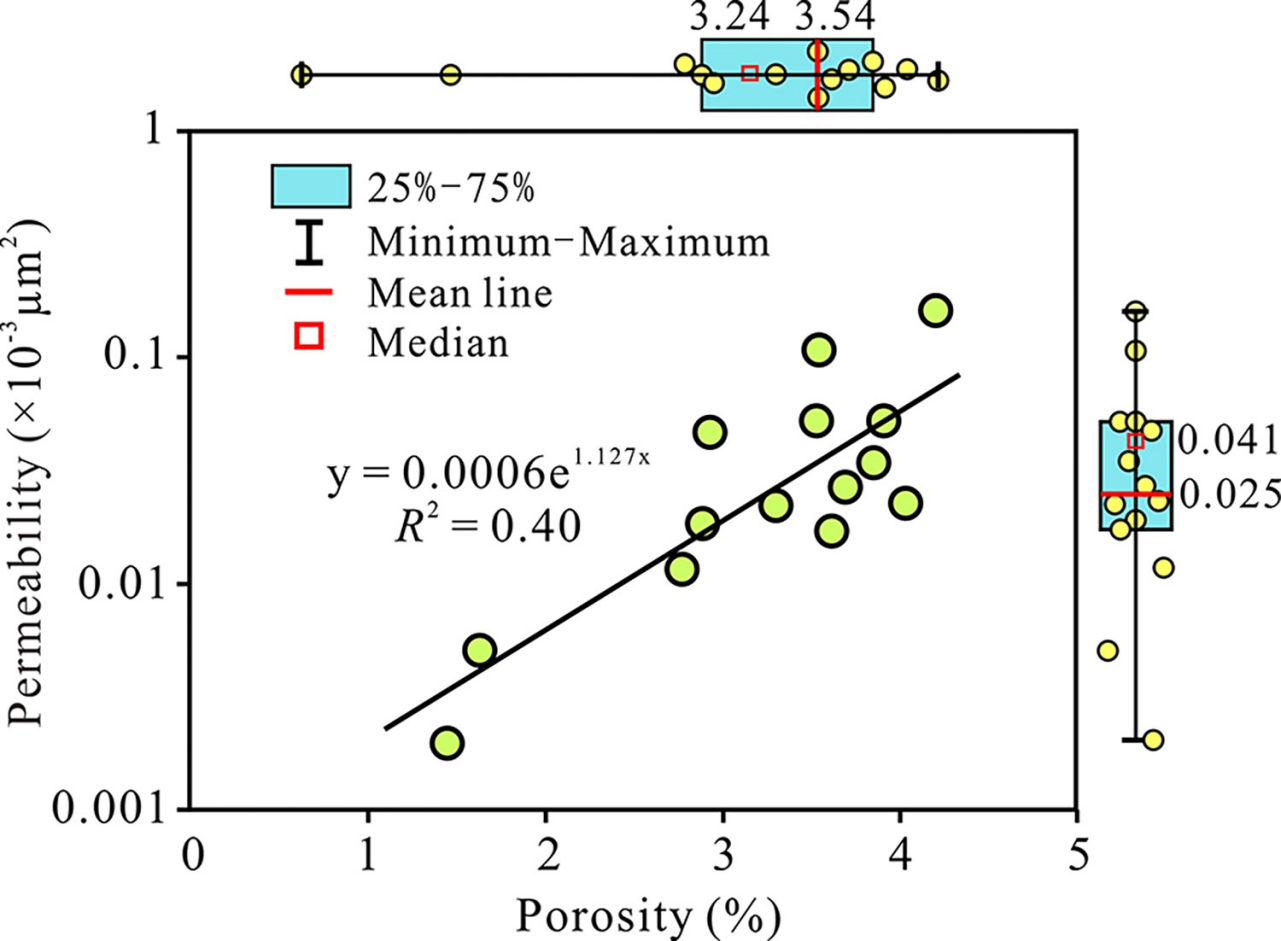
The correlation chart of porosity and permeability of Eh_3_^Ⅸ^ Formation.

The quality of the physical properties reflects the storage capacity of the reservoir and the permeability of fluids in it. The physical properties of the Anpeng deep tight sandstone reservoir are influenced by sedimentation and diagenesis, and generally exhibit low porosity, low permeability to ultra-low permeability characteristics, with strong heterogeneity. The porosity of 14 sandstone samples ranges from 1.46% to 4.21% (averaging 3.24%). The permeability distribution is distributed in 0.002–0.159×10^−3^μm^2^ (averaging 0.041×10^−3^μm^2^). The sandstone reservoirs are mostly low porosity, low permeability–ultra-low permeability tight reservoirs. There is a certain correlation between porosity and permeability, showing a weak exponential correlation (*R*^2^ = 0.40), indicating that the development of pore space in sandstone samples controls the changes in permeability to a certain extent (Fig 3). When the porosity is similar, the permeability difference of multiple sandstone samples is close to an order of magnitude, with strong heterogeneity, indicating that the reservoir permeability is mainly affected by the connectivity of pore throats and pore size distribution [40]. Overall, the Eh_3_^IX^ deep sandstone reservoir is dense and low-permeability, with strong compaction and transformation effects, and poor storage and permeability capacity. The Reservoir Quality Index (*RQI*) and Flow Zone Index (*FZI*) have been widely used to characterize reservoirs with similar pore throat geometric properties (hydraulic units). The higher the *RQI* and *FZI* values of sandstone, the better the reservoir quality and physical properties. The results of Eh_3_^IX^ sandstone samples show that the *RQI* range is 0.012–0.061, with an average value of 0.032, and the *FZI* range is 0.52–1.49, with an average value of 0.88 (Table 1).

### 4.2 Pore-throat types

**Fig 4:**
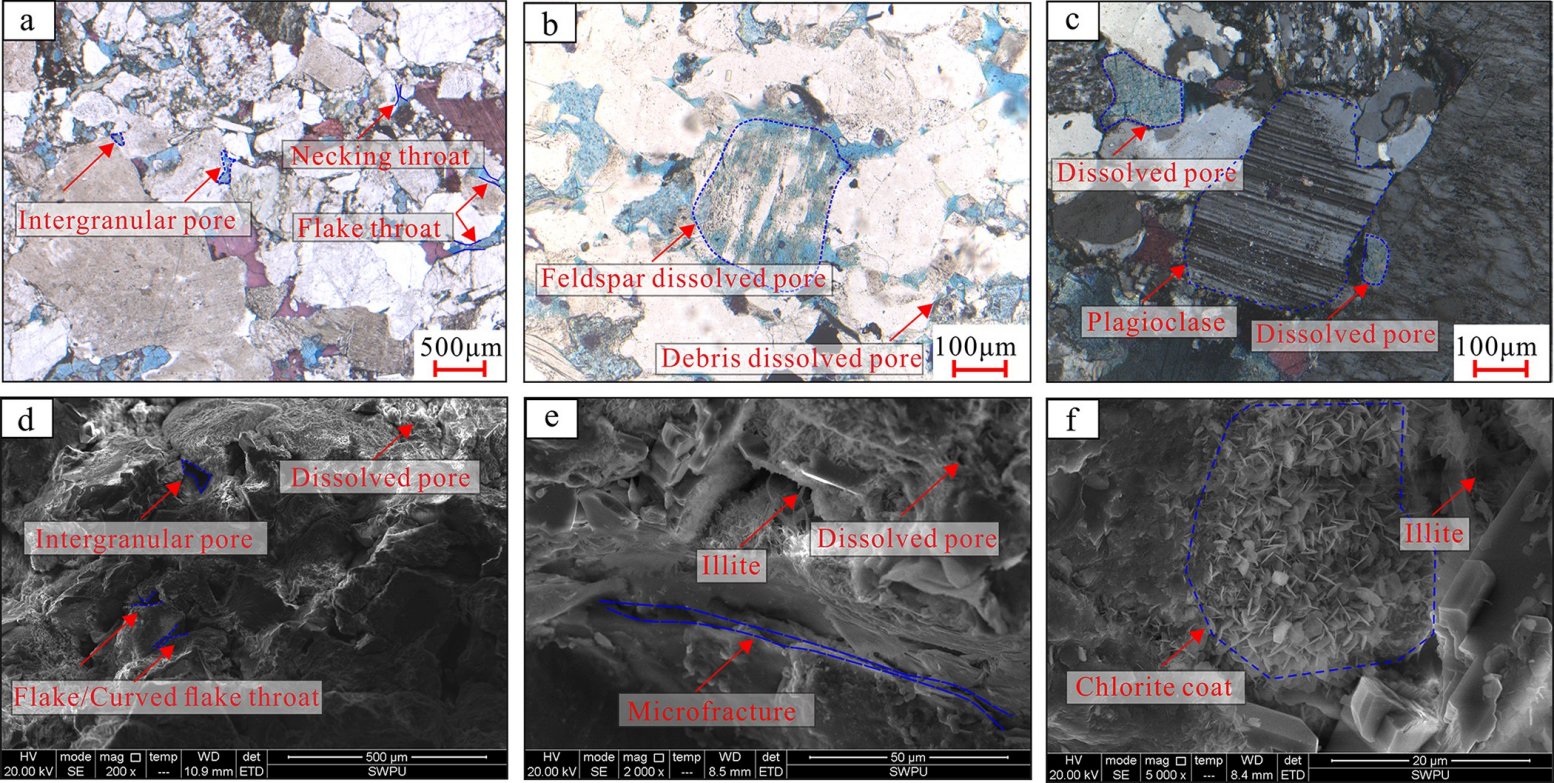
Microscopic characteristics of sandstones in Eh_3_^Ⅸ^ Formation of Anpeng area. (a) Residual intergranular pores and intergranular dissolved pores, flake or curved flake throat, S6, CTS; (b) Common feldspar dissolution pores and lithic dissolved pores, S8, CTS; (c) Feldspar dissolved pores and intergranular micropores, S14, CTS; (d) Intergranular pores and dissolved pores, flake or curved flake throat, S11, SEM; (e) Intergranular bridging illite, development of microcracks, S12, SEM. (f) Petal shaped chlorite adheres to the surface of particles, with enlarged authigenic quartz and the development of microcracks, S8, SEM.

According to the analysis of image data such as cast thin sections and scanning electron microscopy, the porosity of the dense sandstone samples in the target layer of the study area is relatively low (<5%), and the pore development is poor. The pore types are mainly secondary dissolution pores (Fig 4A and 4C) and residual intergranular pores (Fig 4B), followed by heterogenous micropores, intergranular pores, and microcracks (Fig 4D). Among them, dissolution pores are mainly secondary pores with complex pore morphology formed by the dissolution of soluble minerals such as feldspar, rock debris, impurities, and other cementitious materials under the action of formation fluids. The distribution of residual intergranular pores is uneven, with strong heterogeneity, mostly consisting of compacted or cemented residual pores, forming triangles or polygons, with straight pore edges and no obvious dissolution traces. Intergranular pores are pores formed between mineral crystals under the influence of diagenesis, and their pore size is related to the type and production mode of minerals. Usually, the pore throats of bridging illite and illite/montmorillonite are smaller (Fig 4E and 4F), while the intergranular pores of intact kaolin are more developed. Microcracks are intergranular fractures formed by the rupture of rock particles under stress, with a relatively short extension length, mostly in the micrometer range. The types of throats are mainly flake, curved flake, and necked, with poor connectivity between pores and throats, with fine and micro throats dominating. The overall pore throat structure is complex and highly heterogeneous, with a maximum pore throat radius of 0.08–1.24 μm. The average is only 0.45 μm. Different types of pore throat combinations result in significant differences in reservoir physical properties and pore throat structural characteristics.

### 4.3 Pore-throat structure characteristics

**Fig 5:**
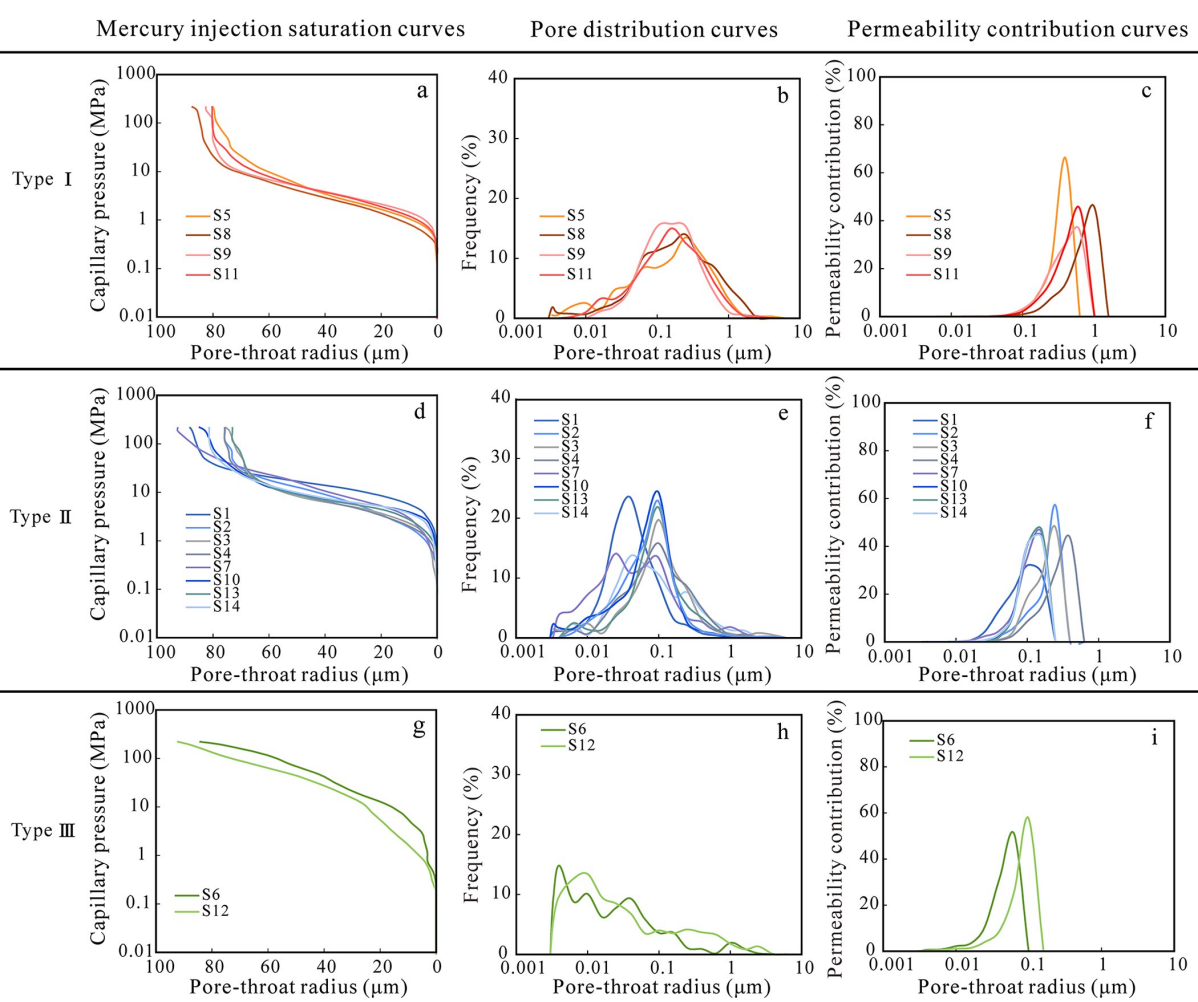
Mercury saturation curves, pore size distribution curves and Permeability contribution curves of Type Ⅰ (a, b, c), Ⅱ (d, e, f), and Ⅲ (g, h, i) samples obtained from HPMI test.

According to the characteristics of capillary pressure curve morphology, pore size distribution, and permeability contribution rate obtained by the HPMI, the pore-throat structures of sandstones are divided into three types, and the physical properties and pore-throat structure characteristics of different types of sandstone are quite different. The pore types of I samples are mainly dissolution pores and intergranular pores, and the middle section of the mercury injection curve has obvious plateau characteristics (Fig 5A). The pore-throat radius is relatively large, and the overall pore size distribution curve shows a unimodal feature. The peak aperture is approximately 0.16 μm (submicron pores) (Fig 5B). The permeability contribution curve of this type of sample exhibits a unimodal characteristic, and the pore-throat radius that contributes to permeability is mainly distributed between 0.3–1.1 μm (sub-micron pores and micro pores) (Fig 5C). The main types of pore space for Type II samples are dissolution pores and intergranular pores. The plateau characteristics of the mercury injection curve have deteriorated, and the curve has overall risen upwards, indicating poor pore throat sorting (Fig 5D). The aperture distribution curve exhibits a mixture of unimodal and bimodal characteristics, with an aperture distribution range mainly ranging from 0.015 to 0.16 μm (submicron and nanopores), with peak pore radius less than 0.1 μm (Fig 5E). The contribution of different pore sizes to permeability varies greatly in Type II samples, and the pore radius corresponding to the peak value decreases (0.06–0.4 μm). And the contribution rate of permeability corresponding to the peak decreases (Fig 5F). The pore types of type III samples are mainly secondary intercrystalline pores and matrix micropores, with a steep and higher position in the middle of the mercury injection curve (Fig 5G). The pore-throat radius is the smallest (nanopore), and the pore size distribution range is 0.004–0.1 μm. The pore size distribution curve shows the three-peak characteristics with the most obvious left peak. The left peak is the micro pore throat (dead pore) with the radius less than 0.01 μm, the middle peak corresponds to the nano pore-throat with the radius less than about 0.03 μm. The right peak corresponds to the sub-nano pore-throat (0.1–0.5 μm), indicating that the pore size distribution of type III samples is mainly nanopores (Fig 5H). The permeability contribution curve of Type III samples is characterized by a single peak. Compared with Type I and II, the pore-throat radius corresponding to the peak is significantly reduced (averaging 0.075 μm), and the peak position is obviously shifted to the left (Fig 5I).

### 4.4 Pore fractal characteristics based on HPMI

**Fig 6:**
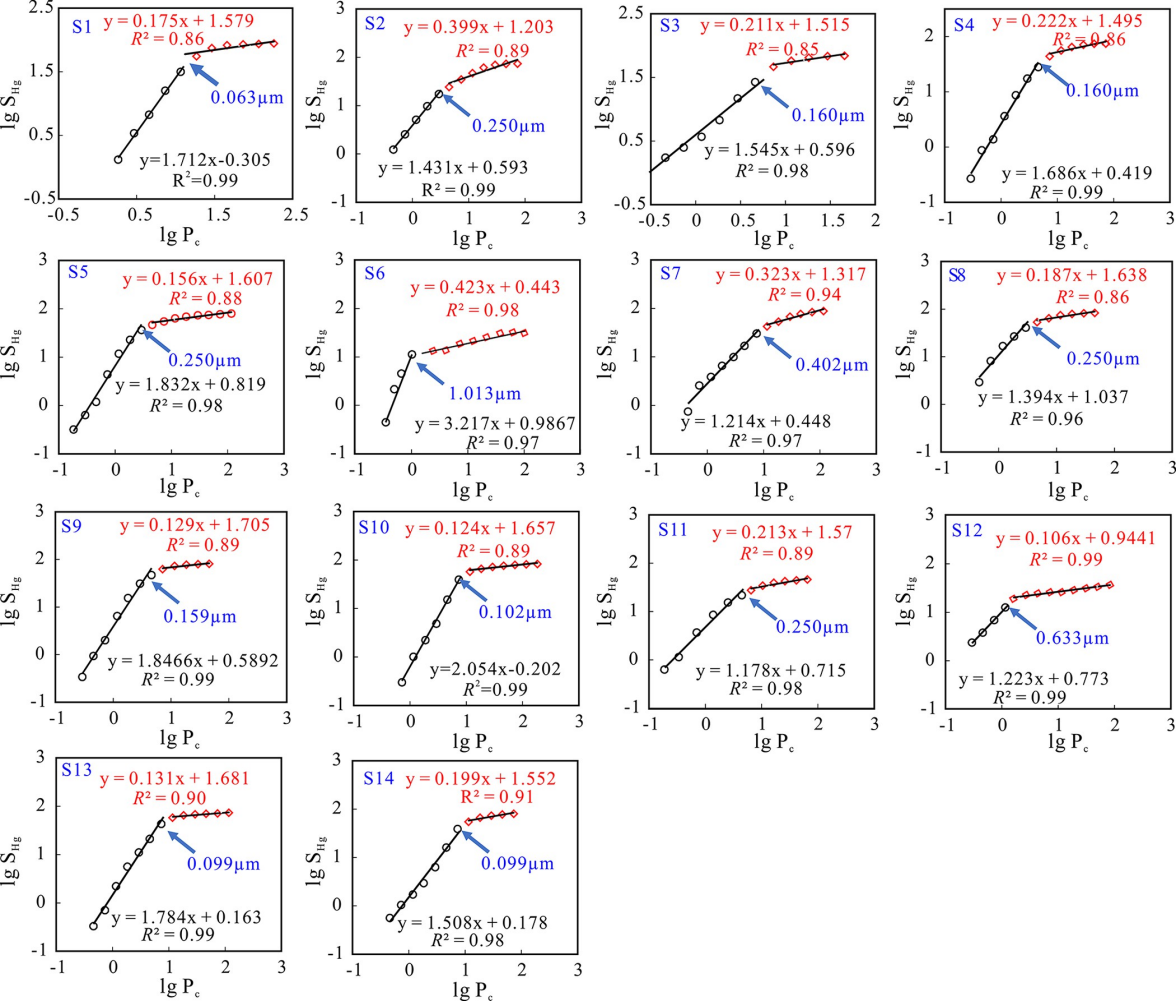
Double logarithmic curves of the mercury inlet pressure (*P*_c_) and the mercury inlet saturation (*S*_Hg)_ of the sandstone samples.

According to the fractal theory, as a typical three-dimensional porous medium, the pore throat structure inside rocks has fractal characteristics within a certain scale range [41]. The cross-section of porous fractal objects is composed of two-dimensional line segments, and the theoretical value of their fractal dimension is between 2 and 3 [42]. The lower limit value 2 indicates a strongly uniform structure or smooth surface of the pore-throat, while the upper limit value 3 indicates a very poor completely heterogeneous structure or an extremely rough pore-throat surface [43]. Based on the capillary pressure curve obtained from HPMI, combined with the mercury saturation model, the double logarithmic diagram was plotted for the mercury saturation (*S*_Hg_) and mercury injection pressure (*P*_c_) of 14 sandstone samples (Fig 6). It can be observed that there are obvious turning points that divide the fractal dimension fitting curve into two segments, and the slope of the fractal curve changes significantly before and after the turning point. The correlation of each fitting curve is strong (*R*^2^ is 0.85–0.99), indicating that the tight sandstone of the Eh_3_^Ⅸ^ Formation has multiple self-similar pore-throat systems. In the logarithmic plot, when the mercury inlet pressure is less than the turning point pressure, it corresponds to a large-scale pore throat; When the mercury inlet pressure is greater than the turning point pressure, it corresponds to a small-scale pore throat. The fractal dimension of large-scale pore throats in sandstone samples is significantly larger than that of small-scale pore throats. The fractal dimension of large-scale pore throats is 3.14–5.22 (averaging 3.72), which is greater than the three-dimensional Euclidean space dimension; The fractal dimension *D*_2_ of small-scale pore throats ranges from 2.12 to 2.52 (averaging 2.25). When the pore-throat radius is greater than the pore-throat radius corresponding to the turning point (*r*_t_), the percentage of the mercury injection amount corresponding to this part of the pore space to the total mercury saturation is the large pore-throat porosity (*Φ*_1_). When the pore-throat radius is less than the *r*_t_, the percentage of mercury injection corresponding to this part of the pore space to the total mercury saturation is small pore-throat porosity (*Φ*_2_). Although under high pressure, mercury cannot enter part of the micropore (*Φ*_3_) in the rock. The comprehensive fractal dimension can better characterize the uniformity of pores, including the average fractal dimension (*D*_a_) and the weighted fractal dimension (*D*_w_). The *D*_a_ is 2.66–3.87 (averaging 2.97). According to the pore-throat volume (*Φ*_1_, *Φ*_2_) corresponding to the *D*_1_ and *D*_2_ intervals, the *D*_w_ (Eq 10) is calculated by weighted average, with a range of 2.19–3.13 (averaging 2.76) (Table 2).

**Table 2:** Fractal dimensions of pore-throat structure obtained from HPMI.

Type	Samples	*Φ* (%)	*K* (×10^−3^ μm^2^)	*r*_t_ (μm)	*D*_1_	*D*_2_	*D*_*a*_	*D*_*w*_	*Φ*_1_ (%)	*Φ*_2_ (%)	*Φ*_3_ (%)
I	S5	3.91	0.052	0.250	3.83	2.16	2.99	2.63	0.89	2.24	0.78
	S8	3.54	0.051	0.250	3.39	2.19	2.79	2.56	0.95	2.15	0.44
	S9	2.93	0.047	0.159	3.85	2.13	2.99	2.77	0.90	1.51	0.52
	S11	3.54	0.107	0.250	3.18	2.21	2.70	2.60	1.13	1.71	0.70
II	S1	3.70	0.027	0.063	3.71	2.18	2.94	3.13	2.03	1.24	0.43
	S2	2.89	0.018	0.250	3.43	2.39	2.92	2.64	0.50	1.69	0.70
	S3	3.61	0.017	0.160	3.54	2.21	2.88	3.03	1.67	1.06	0.88
	S4	3.85	0.035	0.160	3.69	2.22	2.95	2.76	1.08	1.84	0.93
	S7	1.62	0.005	0.097	3.81	2.32	3.07	2.81	0.49	1.01	0.12
	S10	4.04	0.023	0.102	4.05	2.12	3.09	3.02	1.60	1.84	0.61
	S13	3.30	0.022	0.099	3.78	2.13	2.96	3.10	1.41	1.00	0.89
	S14	2.78	0.012	0.099	3.51	2.20	2.85	2.83	1.08	1.18	0.52
III	S6	1.46	0.002	0.633	5.22	2.42	3.87	2.62	0.04	1.19	0.23
	S12	4.21	0.159	0.633	3.22	2.10	2.66	2.19	2.41	1.28	0.52

## 5 Discussion

### 5.1 Pore-throat structure characteristics under alkaline diagenesis

**Fig 7:**
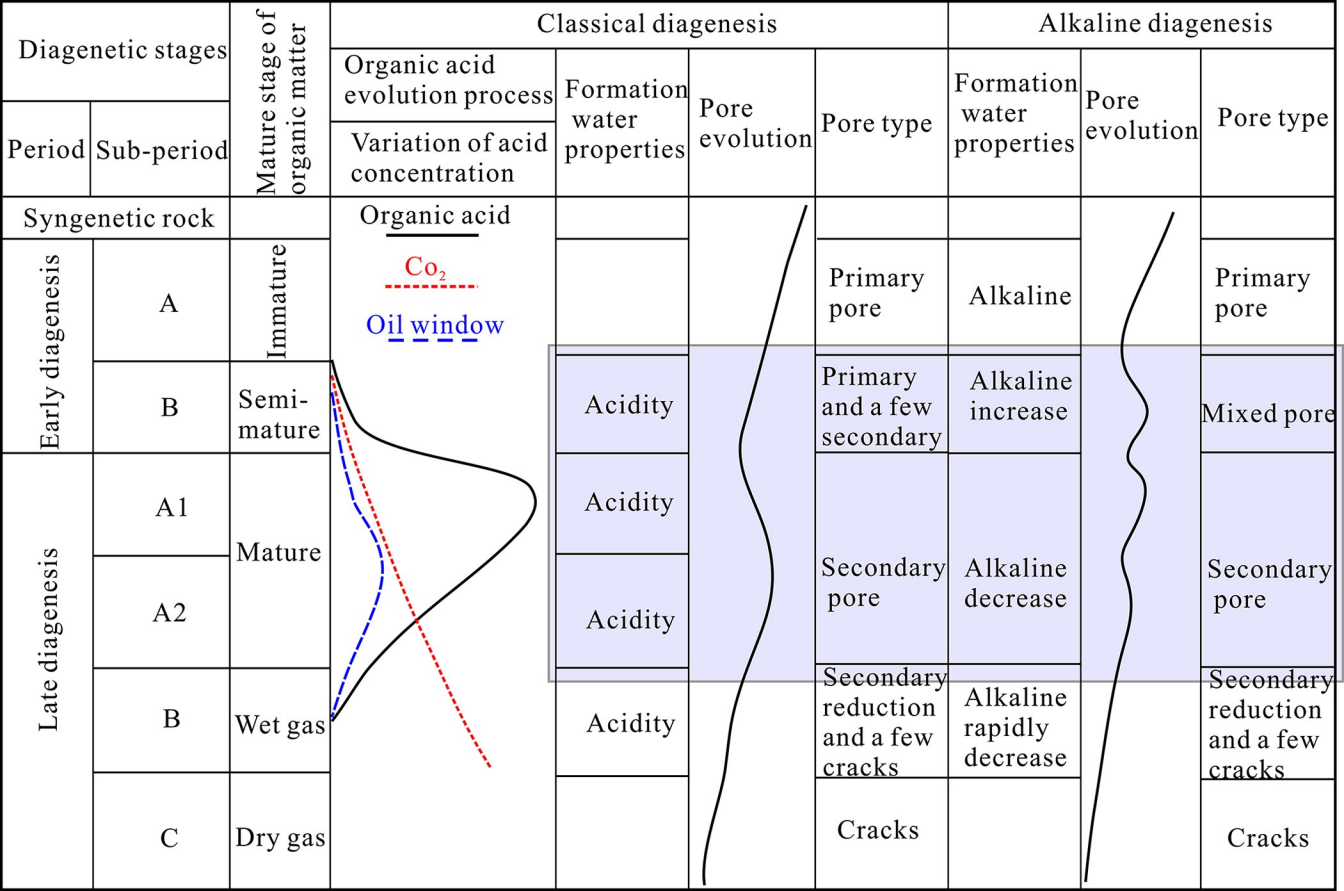
Comparison of the effects of alkaline diagenesis and classical diagenesis on pores.

The porosity distribution of the Eh3IX sandstone reservoir in the research area ranges from 1.5% to 4.2%, with an average of 3.2%. The diagenetic stage is late diagenetic stage B. The reservoir space types are mainly secondary solution pores and intercrystalline micropores, with pore sizes distributed in the nano to submicron range. Compared to the tight sandstone reservoirs in acidic media coal bearing strata, the tight sandstone reservoirs in the study area have poorer physical properties, more complex pore structures, and significant differences in pore size and types. This is because the Anpeng area in the Biyang depression of the Nanxiang Basin was mainly deposited in alkaline lake facies during the third stage of the nuclear period, which determined that the reservoir water was alkaline or strongly alkaline in the early stage of burial. Compared to the tight sandstone reservoirs in acidic coal bearing strata, the Eh3IX tight sandstone reservoirs in the study area exhibit significant differences in diagenetic evolution from classical diagenesis due to alkaline geological environments. Burial diagenesis is an alkaline diagenesis with alkaline water as the background, mainly manifested by significant dissolution of quartz and large-scale enlargement of feldspar, leading to further destruction of intergranular pores. The alkaline diagenesis process leads to significant differences in pore evolution, pore types, and micro heterogeneity of pore-throats at different stages. These differences are specifically reflected in the poorer physical properties, smaller pore sizes (mainly nanometer and submicron), poorer pore throat connectivity, and pore spaces mainly consisting of secondary dissolution pores and intergranular micropores in the tight sandstone reservoirs of the alkaline strata in the study area. In addition, due to the burial depth of the lower segment of the third nuclear power plant exceeding 3300m and the strong mechanical compaction effect, the pore space of the reservoir is compacted, resulting in poor pore development and a denser reservoir. The main manifestation is that the early diagenetic stage B is the main development period of pores, while the late diagenetic stage A is generally not conducive to the development of reservoir space (Fig 7). According to fractal geometry theory, the fractal dimension of three-dimensional Euclidean space objects cannot theoretically exceed 3, indicating that large-scale pores and throats do not have fractal structural characteristics within this size range. Therefore, the differences in pore throat structure caused by different diagenetic processes play an important role in fractal characteristics and heterogeneity. Therefore, it is necessary to study the pore-throat structure characteristics of tight sandstone in alkaline formations to clarify the differences in pore throat structure characteristics between terrestrial alkaline formations and terrestrial acidic coal bearing formations in tight sandstone reservoirs.

### 5.2 Binary pore-throat system in tight sandstone

**Fig 8:**
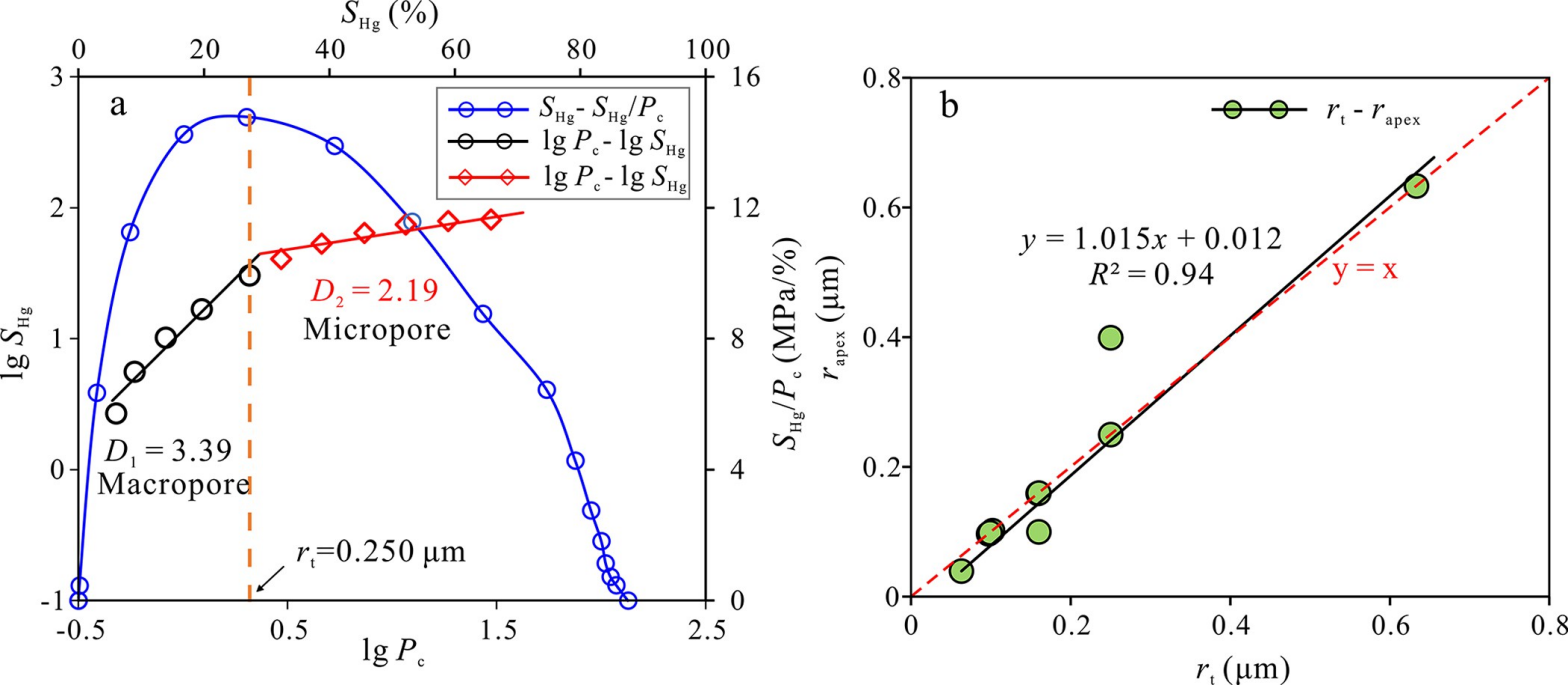
Fractal characteristics of pore throats at different scales. (a) The superposition diagram of Pittman curve and fractal curve of typical sandstone sample (S8); (b) The correlation between the turning point radius of fractal curves (*r*_t_) and the vertex radius of Pittman curves (*r*_apex_) of 14 sandstone samples.

The contribution of various pore-throat scales (geometric shape, pore size and distribution) to the porosity and permeability of sandstone reservoirs differs significantly [44]. The fractal dimension can provide a theoretical foundation for revealing the geometric and multi-scale characteristics of heterogeneous porous media in tight sandstone reservoirs. The Pittman curve is plotted using mercury saturation divided by pressure as the vertical axis and mercury saturation as the horizontal axis (Fig 8A). The pore throat radius (*r*_apex_) corresponding to the Pittman curve’s apex represents a watershed marking the connectivity transition of the rock pore-throat system. The higher the *r*_apex_ value, indicating better sorting and more concentrated development of the large pore-throats with good connectivity [45, 46]. By superimposing the *S*_Hg_/*P*_c_—*S*_Hg_ relationship graph with the fractal curve, it was observed that the vertices determined by Pittman align with the turning points of the fractal curve, and the vertex radius closely matches the turning radius (*R*^2^ = 0.94) (Fi 8b). Therefore, the *r*_t_ is a key node for dividing the reservoir space into large and small pore throats.

The transition from large pore-throats to small pore-throats signifies to a gradual transition in pore types from being dominated by intergranular pores and dissolved pores to being dominated by dissolved pores and intercrystalline micropores. The distribution range of *r*_t_ is 0.06–0.89 μm (averaging 0.25 μm). The distribution characteristics of the turning point radius of the fractal dimension fitting curve of rock samples are quite different, indicating significant heterogeneity in the pore throat structures of tight sandstone.

**Fig 9:**
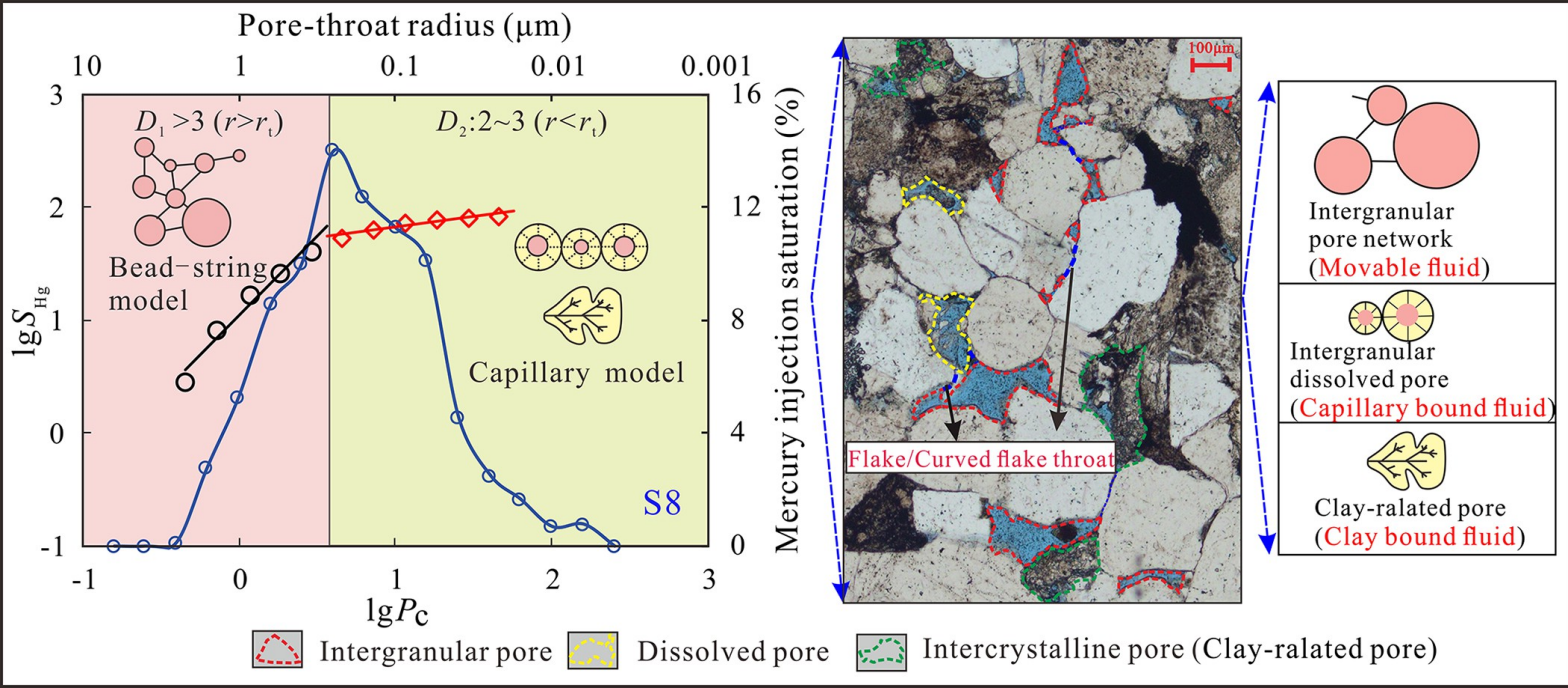
Binary pore structure model of tight sandstone.

According to fractal theory, only within a certain characteristic length range, the fractal dimension can effectively characterize the heterogeneity of pore-throat space in porous media (rock). At this point, the three-dimensional structure of the pore-throat satisfies self-similarity and scale invariance. Assuming the fractal dimension of the pore shape is n, this implies that as the pore radius is scaled by a certain factor, its volume is also scaled by the nth power of the factor. When an object undergoes bi-directional scaling, the fractal dimension is 2. When the object is three-dimensional scaling, the fractal dimension is 3. When the object is three-dimensional non-proportional expansion, the fractal dimension exceeds 3 [47]. Based on the HPMI experiment and the non-wetting fractal dimension calculation model, the pore-throat structure is simplified into a set of capillaries with different radii. Theoretically, the fractal dimension of the three-dimensional pore space inside the rock range between 2 and 3. However, tight sandstone exhibits small pore size and complex pore-throat structure, with the fractal dimension of large pore-throats often exceeding 3. This suggests that the actual pore-throat structure of ultra-low permeability tight sandstone is more complex than the simplified capillary model. Through the observation of SEM and CTS images (Fig 4), it is found that due to the complex sedimentary and diagenesis of the Eh_3_^Ⅸ^ formation in the study area, the tight sandstone develops large pores such as intergranular pores, dissolved pores and a small number of micro-fractures, as well as small pores such as intercrystalline pores and clay-related pores. There are significant differences in pores at different scales. When the large pores are connected to the wide throats (flake throats) or there are micro-cracks, the characteristic length of the pore-throat is quite different, and the pore-throat structure is similar to the bead-string model. The average fractal dimension of large-scale pores is 3.72, and the pore-throat structure exhibits a three-dimensional non-proportional extension, which makes the slope of the fractal dimension fitting curve increase rapidly, resulting in abnormal fractal dimension and not meeting the fractal geometric relationship in the non-wetting phase model. When the small pore-throat is connected with the narrow throats (necked, point shaped throats), the pore radius is approximately equal to the throat radius, which can be simplified into the capillary model. The average fractal dimension of this part of the pore-throat is 2.22, and the pore-throat has the characteristics between two-dimensional and three-dimensional extension, which conforms to the fractal geometry theory (Fig 9). Previous studies have shown that based on the assumption that the pore-throat space is an ideal cylindrical shape, the fractal dimension can be calculated based on the fractal relationship between mercury saturation and mercury injection pressure. Therefore, only when the pore-throat radius is close, the fractal dimension calculated based on the non-wetting phase model can effectively represent the heterogeneity characteristics of the pore-throat structure. However, for micro-fractures, large pore throats with large difference in pore and throat radius, due to the significant difference in characteristic length and geometric shape, the pore space cannot satisfy the self-similarity in a certain scale. At the same time, the fractal dimension of pore space greater than 3 can also serve as a good indicator to determine whether micro-fractures are developed inside the rock or to characterize the degree of micro-fracture development.

### 5.3 Relationship between fractal dimension and reservoir physical properties / pore-throat structure

**Fig 10:**
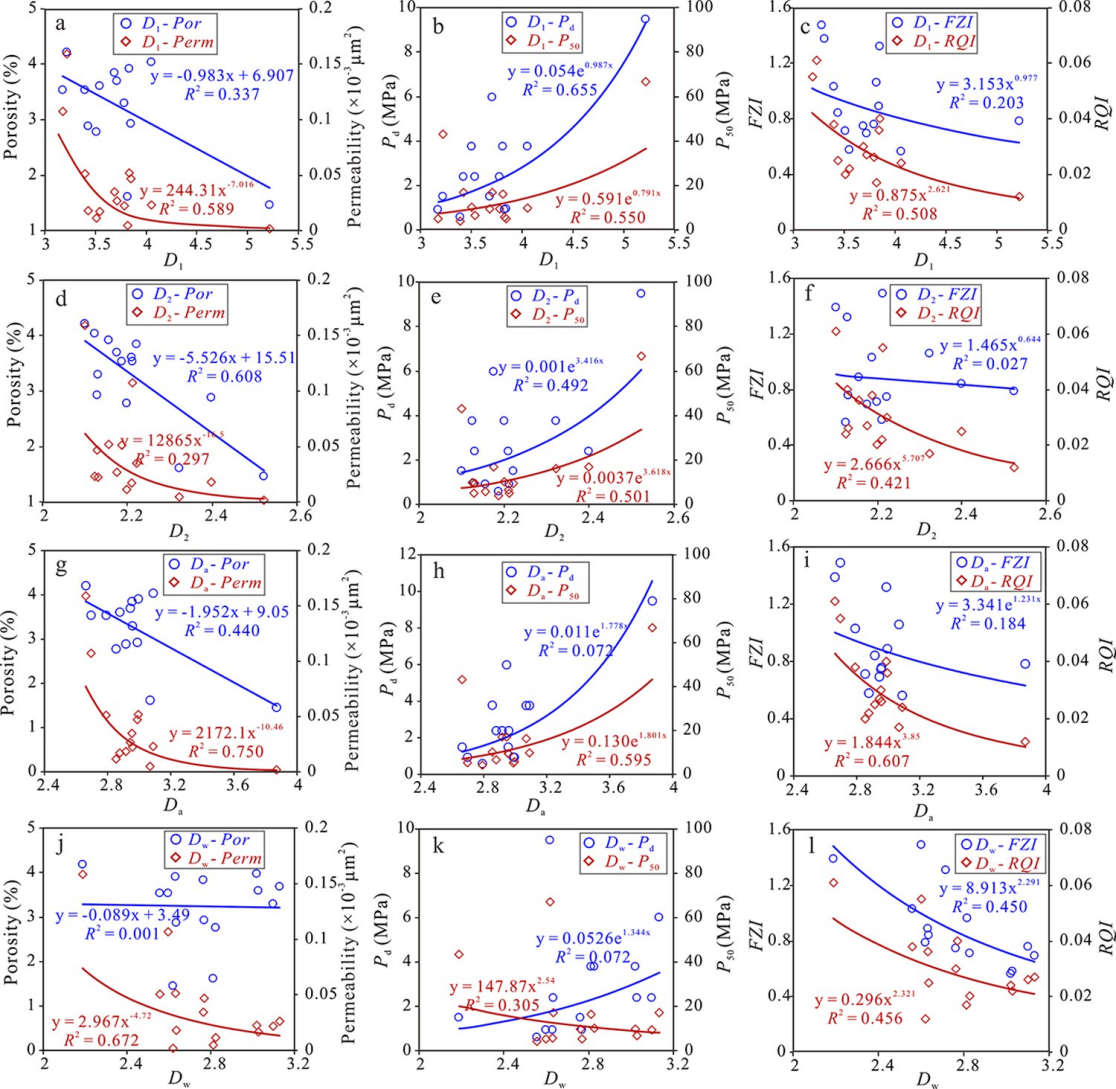
Relationship between different fractal dimensions (*D*_1_, *D*_2_, *D*_a_, and *D*_w_) and reservoir physical properties, pore-throat structure, and reservoir quality parameters.

The theory of fractal geometry indicates that fractal dimension can be used to evaluate the complexity and irregularity of pore-throat structures. The increase of fractal dimension often reflects more complex pore-throat structure, and the reservoir permeability is reduced, which makes the reservoir quality worse [48, 49]. Based on the correlation analysis between fractal dimension (*D*_1_, *D*_2,_ *D*_a_, and *D*_w_) and reservoir physical properties (*Φ*, *K*), pore-throat structure parameters (*P*_d_, *P*_50_) and reservoir quality parameters (*FZI*, *RQI*), the effectiveness of fractal dimension in evaluating pore-throat structure and reservoir quality is clarified. The fractal dimension (*D*_1_) of large-scale pore throats has a poor correlation with porosity (*R*^2^ = 0.34), and a significant power correlation with permeability (*R*^2^ = 0.59), indicating that a small number of large-scale pore-throats play a major role in permeability. *D*_1_ has a good correlation with pore structure parameters. With the increase of *D*_1_, the reservoir quality significantly deteriorates. *D*_1_ has a good correlation with *FZ*I, but a weak exponential correlation with *RQI* (*R*^2^ = 0.20). This may be because *RQI* only reflects the physical properties of the reservoir, while *FZI* is a comprehensive index that characterizes the physical properties and pore-throat structure of the reservoir (Fig 10A–10C). The fractal dimension (*D*_2_) of small-scale pore throats has a good correlation with porosity (*R*^2^ = 0.61), but has a weak correlation with permeability (*R*^2^ = 0.30). This may be because the fluid flow characteristics in porous media are mainly controlled by the characteristic length. The Eh_3_^IX^ Formation tight sandstone reservoir in the study area has many small pore-throats, and the pore-throats connectivity is poor, which affects the fluid mobility of the reservoir, resulting in that the fractal dimension of small pore-throats cannot effectively characterize the permeability of the entire pore-throat space. *D*_2_ has a good correlation with pore-throat structure parameters, but has a poor correlation with *FZI* (*R*^2^ = 0.32). There is basically no correlation between *D*_2_ and *RQI*, indicating that *D*_2_ cannot characterize the overall physical properties and pore-throat structure characteristics of sandstone reservoirs (Fig 10D–10F). Due to the binary pore-throat structure characteristics of tight sandstone, *D*_1_ and *D*_2_ cannot characterize the overall pore-throat structure and the reservoir physical property quality. Therefore, the comprehensive fractal dimensions *D*_a_ and *D*_w_ are analyzed. The correlation between *D*_a_ and various characteristic parameters is good, among which *D*_a_ has the best correlation with *K*, *P*_50_, and *FZI*, and the correlation coefficients are 0.75, 0.60, and 0.61, respectively, indicating that *D*_a_ can effectively characterize the overall pore-throat heterogeneity and the quality of reservoir physical properties (Fig 10G–10I). With the increases of *D*_a_, the physical properties and the quality of reservoir become worse, and the pore-throat structure becomes more complex. From Type I to Type III sandstone, the larger the *D*_a_, the stronger the heterogeneity of the pore-throat structure. Comparing the correlation between the two fractal dimensions and various parameters, *D*_w_ has poor correlation with *P*_d_, *P*_50_, and *Φ*. which may be because the physical meaning of *D*_w_ depends more on the pore-throat size distribution (Fig 10J–10L).

### 5.4 Effect of different scales pore-throat on reservoir physical properties

**Fig 11:**
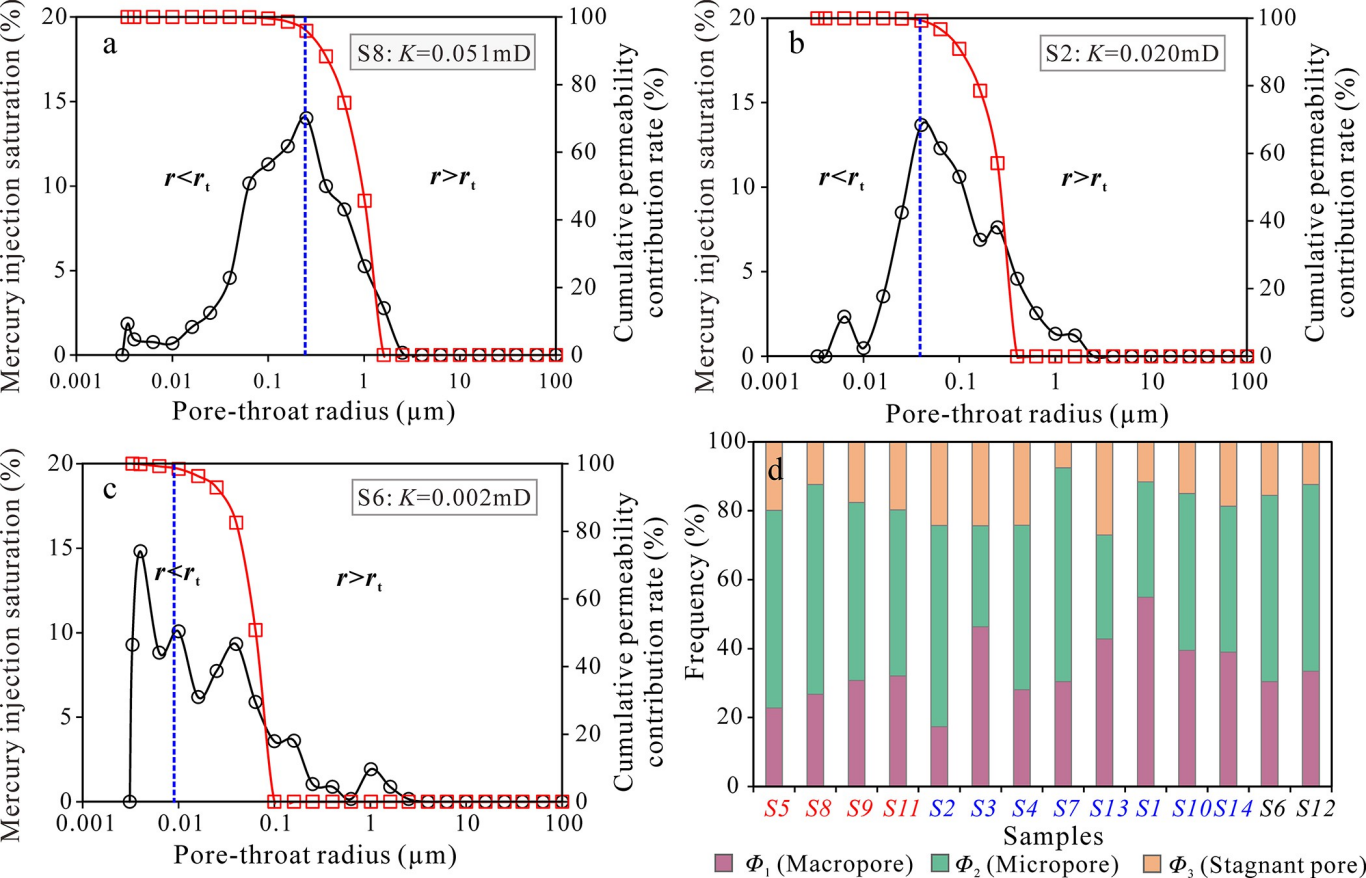
Distribution characteristics of pore-throats of different scales. (a-c) The distribution relationship between pore-throat size and mercury saturation and permeability contribution value for three types of typical sandstone samples (S8, S2, and S6) from type I to type Ⅲ; (d) Pore-throat volume distribution characteristics of sandstone samples at different scales.

Many studies have confirmed that large pore-throats in tight sandstone reservoirs often determine the permeability of the reservoir, while pore volume is more contributed by small pore-throats. Three typical sandstone samples (S8, S2, and S6) were selected for comparative analysis of their pore size distribution and cumulative permeability contributions. As the physical properties of the three types of sandstones deteriorate and their pore structures become more complex, *r*_t_ decreases gradually, and the pore-throat scale that plays a major role in permeability changes from submicron pores to nanopores. The turning point radius rt of the fractal curve of type I and II sandstones corresponds to the peak value of the pore size distribution, and corresponds to the highest value of the cumulative permeability contribution (about 99%). However, due to the complex pore-throat structure of type III sandstones, the pore size distribution curve shows multi-peak characteristics, and *r*_t_ corresponds to the middle peak of pore size distribution. At this point, the cumulative permeability contribution is approximately 99%. The overall performance shows that as the pore-throat radius decreases, the permeability contribution gradually weakens, and the cumulative permeability contribution reaches the peak (Fig 11A–11C). This indicates that the pore-throat size and distribution characteristics are the key factors in controlling reservoir space size and fluid flow capacity, which directly influencing the quality of reservoir physical properties. The proportion of large pore-throat volume is an important factor affecting reservoir physical properties, whereas small pores and micropores mainly affect the volume of effective pores and have little impact on fluid seepage. This is due to a thin film of bound water is attached to the pore-throat surfaces within the hydrophilic tight sandstone in the study area, which increases the flow resistance of the fluid in the small pores and micropores, resulting in ineffective fluid migration. The contribution of large pore-throats to sandstone permeability is significant, which is the dominant factor in reservoir physical properties. More developed small pore-throats and micropores that mercury cannot enter will be detrimental to reservoir quality and seepage dynamics.

For ultra-low permeability sandstone reservoirs, different pore-throat scale division standards result in significant differences in defining the reservoir pore size, which affects the calculation and prediction of reservoir physical parameters. Statistical analysis was conducted on the pore volumes corresponding to the large pores, small pores, and micropores at the turning points of fractal curves (Table 2), and the correlation between the pore volume and the reservoir physical properties is analyzed. The sandstone reservoir mainly develops small pore-throats, with slightly less proportion of large pore-throats and the least proportion of micropores volume. The average volume proportions of large pores, small pores and micropores are 33.9%, 48.2%, and 17.9%, respectively. The sandstone reservoir in the study area has a deep burial depth and a high degree of diagenesis evolution. It has evolved to the late diagenesis stage and exhibits strong compaction, pressure dissolution, and cementation. The strong compaction and pressure dissolution are manifested in the line contact or concave-convex contact between quartz particles, and the strong cementation is manifested in the development of carbonate cements such as calcite and iron-bearing dolomite. These diagenetic processes directly or indirectly damage the primary macropores between particles, resulting in less preservation of macropores and reduced porosity. The dissolution of the formation acidic fluid and the unstable clastic particles in the sandstone increases the porosity, but the pores show that the roughness is large, the pore geometry is complex, and the pore throat structure is highly heterogeneous. The acidic fluid in the formation dissolves with unstable debris particles in sandstone, causing an increase in porosity. However, the pores show high roughness, complex pore geometries, and strong heterogeneity in pore-throat structure. Therefore, the volume of large pores and micropores in sandstone reservoirs varies greatly, while the volume of small pores is relatively stable (Fig 11D).

### 5.5 Permeability estimation model

Permeability is an important parameter that characterizes the seepage characteristics of sandstone reservoir, which is closely related to the pore-throat characteristics. Previous studies have shown that the permeability in tight reservoirs is mainly controlled by pore-throat size and pore-throat type, and the influence of pore-throat radius on permeability is greater than porosity. When the porosity of the tight sandstone reservoir in Eh_3_^Ⅸ^ Formation is close, the corresponding permeability is different by one or more orders of magnitude, resulting in a weak correlation between porosity and permeability (*R*^2^ = 0.40). This indicates that the permeability of sandstone reservoirs in the study area is not only controlled by porosity, but also influenced by pore-throat network characteristics and micro-fractures. It is crucial to select reasonable parameters to describe the pore-throat size, distribution, and connectivity of different rock samples in order to effectively characterize permeability. Based on mercury injection experiments, the characteristic lengths of rock pore- throats (*r*_a_, *r*_50_, and *r*_d_) can be obtained. These parameters are positively correlated with permeability, but the correlation is weak. On this basis, Winland proposed an empirical formula (Eq 11) between permeability, porosity, and characteristic length, where the characteristic length is the pore radius *r*_35_ corresponding to a mercury saturation of 35%. Pittman proposed a characteristic length that takes into account the fluid permeability of the reservoir, namely the vertex radius *r*_apex_ of the Pittman curve.

logr=c+alogK+blogϕ (11)

where *r* is the characteristic length (μm); *K* is the permeability (×10^−3^μm^2^); *Φ* is porosity (%); a, b, and c are constants.

**Fig 12:**
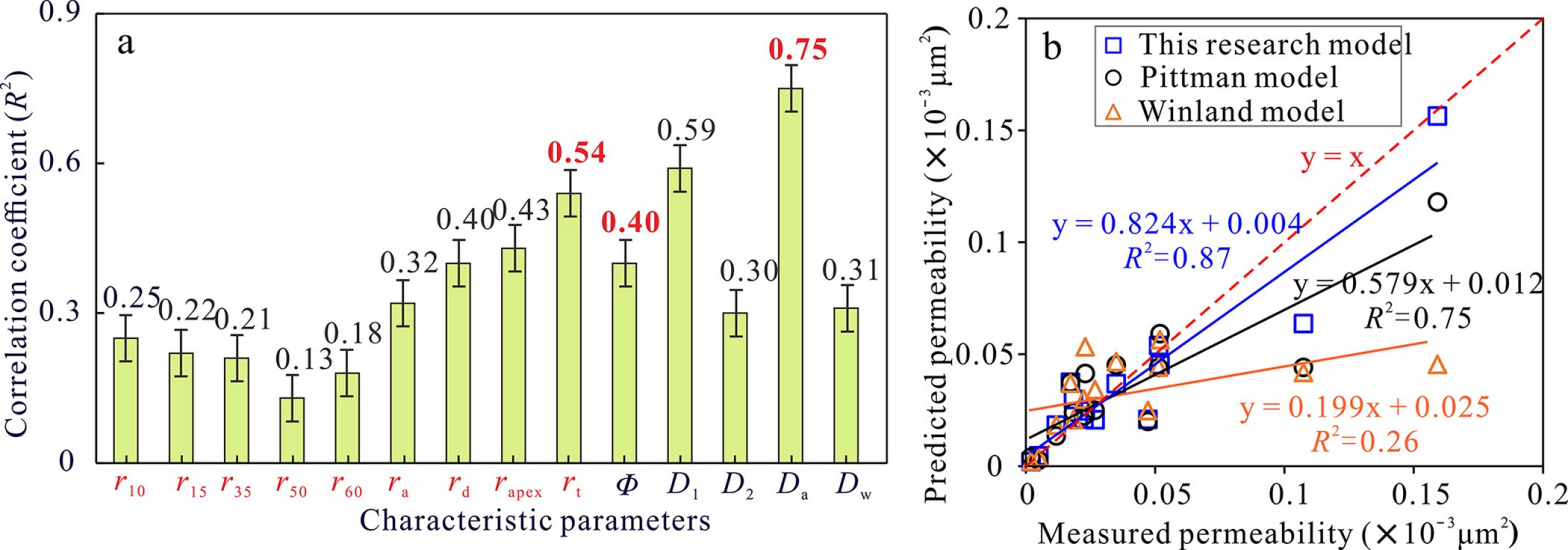
Permeability estimation model. (a) Correlation between characteristic parameters and permeability; (b) The relationship between predicted permeability and measured permeability.

Based on the mercury injection test data, the permeability, porosity and different characteristic lengths (*r*_10_, *r*_15_, *r*_35_, *r*_50_, *r*_60_, *r*_a_, *r*_d_, *r*_apex_) are respectively subjected to multiple regression to obtain the corresponding permeability prediction expressions, and the calculated permeability values were compared with the measured permeability values. The result show that the above permeability regression model has poor estimation performance and cannot be applied to accurately calculate the permeability of tight sandstone reservoirs in the study area (Table 3). Based on the previous analysis, the fractal characteristics have a significant impact on the permeability, and the different scale pore-throats are also key factors to control the permeability. Therefore, through theoretical analysis and multiple model calculations, the three parameters of *Φ*, *D*_a_ and *r*_t_ are finally selected for multiple regression estimation of permeability (Fig 12A). The permeability estimation model proposed in this study is more accurate (*R*^2^ = 0.85). The measured permeability and the predicted permeability are on the straight line with *y* = *x*, and the coincidence rate is as high as 0.87, which is significantly better than other models (Fig 12B). It shows that the regression model can effectively estimate the permeability of ultra-low permeability tight sandstone reservoirs and provide ideas for logging calculation of tight sandstone reservoir parameters.

**Table 3:** Permeability estimation models with different characteristic parameters.

Characteristic parameters	Permeability fitting formulas	Correlation coefficients (*R*^2^)
*φ*, *r*_10_	$\mathrm{lg}K=2.327\mathrm{lg}\phi +0.753\mathrm{lg}r_{35}-2.378$	0.64
*φ*, *r*_15_	$\mathrm{lg}K=2.263\mathrm{lg}\phi +0.718\mathrm{lg}r_{35}-2.271$	0.68
*φ*, *r*_35_	$\mathrm{lg}K=3.105\mathrm{lg}\phi +0.337\mathrm{lg}r_{35}-2.884$	0.67
*φ*, *r*_50_	$\mathrm{lg}K=2.871\mathrm{lg}\phi +0.271\mathrm{lg}r_{50}-2.932$	0.65
*φ*, *r*_60_	$\mathrm{lg}K=1.854\mathrm{lg}\phi +0.701\mathrm{lg}r_{35}-1.584$	0.61
*φ*, *r*_d_	$\mathrm{lg}K=2.077\mathrm{lg}\phi +0.663\mathrm{lg}r_{d}-2.315$	0.72
*φ*, *r*_a_	$\mathrm{lg}K=2.851\mathrm{lg}\phi +0.120\mathrm{lg}r_{a}-2.840$	0.61
*φ*, *r*_apex_		$\mathrm{lg}K=3.105\mathrm{lg}\phi +0.337\mathrm{lg}r_{35}-2.884$	0.69
*φ*, *r*_t_	$\mathrm{lg}K=2.964\mathrm{lg}\phi +0.506\mathrm{lg}r_{t}-2.678$	0.77
*φ*, *r*_t_, *D*_a_	$\mathrm{lg}K=1.850\mathrm{lg}\phi +0.557\mathrm{lg}r_{t}-0.736D_{a}+0.181$	0.85

## 6 Conclusions

1. The Eh_3_^IX^ tight sandstone reservoir in the study area has significant differences in diagenetic evolution from classical diagenesis due to alkaline geological environments, mainly manifested by significant dissolved of quartz and large-scale enlargement of feldspar, leading to further destruction of intergranular pores and poor pore development. The pore types of sandstone reservoirs are mainly dissolved pores, residual intergranular pores, and intercrystalline micropores, with throats mainly in the form of flake /curved flake and necking shapes. The micro pore-throat structure of the reservoir has multi-scale distribution characteristics, mainly developing nano and submicron pores.
2. The pore-throat structure of tight sandstone based on mercury saturation model has segmented multifractal characteristics, which is reflected in the clear turning point on the fractal dimension curve (lg*P*_c_-lg*S*_Hg_). The turning point of fractal dimension divides the pore-throat structure of tight sandstone into large-scale pore-throats with good connectivity (reticular or beaded pore-throats) and small-scale pore-throats with poor connectivity (dendritic or capillary pore-throats), indicating that tight sandstone has binary pore structure characteristics.
3. The geometric shape and pore size corresponding to different scales of pore-throats are important factors affecting the fractal characteristics and heterogeneity of pore-throat structures. The compaction and cementation effects of small-scale pore-throats (intergranular dissolved pores or intercrystalline micropores paired with necking throats) are strong, the intergranular pores are reduced, the heterogeneity is weak, and the average fractal dimension is 2.22. The dissolution of large-scale pore-throats (dissolved macropores and composite pores paired with flake/curved flake throats) is strong, and the pore-throat radius is large, but the pore morphology is irregular and the heterogeneity is strong. The average fractal dimension is 3.73, which does not meet the fractal relationship in the non-wetting phase model.
4. There are many small pores and micropores in sandstone reservoirs, and their pore volume is significantly positively correlated with the total porosity of the rock, but their contribution to permeability is relatively low. The volume proportion of large-scale pore-throats is an important factor in controlling the physical properties and quality of reservoirs, and the permeability is mainly contributed by the large pore-throats. The wide distribution of pore-throat size, strong heterogeneity, and large tortuosity are the main reasons for the strong heterogeneity of reservoir physical properties and poor correlation between porosity and permeability. Based on the above reasons, it is determined that the parameters of turning point radius, average fractal dimension, and porosity have the best correlation with permeability, and are the optimal pore-throat characteristic parameters for characterizing the seepage characteristics of low-permeability tight sandstone. Establishing a multiple regression estimation model for permeability, with a compliance rate of up to 87%.
